# Nitric Oxide Mobilizes Intracellular Zn^2+^ via the GC/cGMP/PKG Signaling Pathway and Stimulates Adipocyte Differentiation

**DOI:** 10.3390/ijms23105488

**Published:** 2022-05-14

**Authors:** Chien-Wei Chen, Luen-Kui Chen, Tai-Ying Huang, De-Ming Yang, Shui-Yu Liu, Pei-Jiun Tsai, Tien-Hua Chen, Heng-Fu Lin, Chi-Chang Juan

**Affiliations:** 1 College of Human Development and Health, National Taipei University of Nursing and Health Sciences, Taipei 112303, Taiwan; gogozipper130@gmail.com; 2 Institute of Physiology, College of Medicine, National Yang Ming Chiao Tung University, Taipei 112304, Taiwan; luenkui0506@gmail.com (L.-K.C.); c11027065@gmail.com (T.-Y.H.); medium3927@hotmail.com (S.-Y.L.); 3 Institute of Biophotonics, College of Biomedical Science and Engineering, National Yang Ming Chiao Tung University, Taipei 112304, Taiwan; dmyang@vghtpe.gov.tw; 4 Department of Medical Research, Taipei Veterans General Hospital, Taipei 112201, Taiwan; 5 Institute of Anatomy and Cell Biology, College of Medicine, National Yang Ming Chiao Tung University, Taipei 112304, Taiwan; pjtsai@nycu.edu.tw (P.-J.T.); chen_th@vghtpe.gov.tw (T.-H.C.); 6 Department of Critical Care Medicine, Taipei Veterans General Hospital, Taipei 112201, Taiwan; 7 Trauma Center, Taipei Veterans General Hospital, Taipei 112201, Taiwan; 8 Division of General Surgery, Department of Surgery, Taipei Veterans General Hospital, Taipei 112201, Taiwan; 9 Division of Trauma, Department of Surgery, Far-Eastern Memorial Hospital, New Taipei City 220216, Taiwan; 10 Graduate Institute of Medicine, Yuan Ze University, Taoyuan 320315, Taiwan; 11 Department of Education and Research, Taipei City Hospital, Taipei 103212, Taiwan

**Keywords:** zinc, nitric oxide, guanylyl cyclase, protein kinase G, differentiation, adipocyte, obesity

## Abstract

Plasma and tissue zinc ion levels are associated with the development of obesity. Previous studies have suggested that zinc ions may regulate adipocyte metabolism and that nitric oxide (NO) plays a pivotal role in the regulation of adipocyte physiology. Our previous study showed that chronic NO deficiency causes a significant decrease in adipose tissue mass in rats. Studies also suggested that zinc ions play an important modulatory role in regulating NO function. This study aims to explore the role of zinc ions in NO-regulated adipocyte differentiation. We hypothesized that NO could increase intracellular Zn^2+^ level and then stimulate adipocyte differentiation. ZnCl_2_ and the NO donor, NONOate, were used to explore the effects of Zn^2+^ and NO on adipocyte differentiation. Regulatory mechanisms of NO on intracellular Zn^2+^ mobilization were determined by detection. Then, Zn^2+^-selective chelator TPEN was used to clarify the role of intracellular Zn^2+^ on NO-regulated adipocyte differentiation. Furthermore, the relationship between adipocyte size, Zn^2+^ level, and NOS expression in human subcutaneous fat tissue was elucidated. Results showed that both ZnCl_2_ and NO stimulated adipocyte differentiation in a dose-dependent manner. NO stimulated intracellular Zn^2+^ mobilization in adipocytes through the guanylate cyclase (GC)/cyclic guanosine monophosphate (cGMP)/protein kinase G (PKG) pathway, and NO-stimulated adipocyte differentiation was Zn^2+^-dependent. In human subcutaneous adipose tissue, adipocyte size was negatively correlated with expression of eNOS. In conclusion, NO treatment stimulates intracellular Zn^2+^ mobilization through the GC/cGMP/PKG pathway, subsequently stimulating adipocyte differentiation.

## 1. Introduction

Obesity is a global public health problem that no country has perfectly resolved. An epidemiological study reported that more than 650 million adults are estimated to be obese, a figure that, alarmingly, has nearly tripled since 1975 [1]. Obesity adversely affects almost every physiological system of the body, and can significantly complicate conditions including hypertension [2], cardiovascular disease, systemic inflammation, and type II diabetes [3,4]. Clinicians and scientists have voiced concerns that complex health complications are caused by excessive body weight. It is well known that obesity is caused by the hypertrophy or hyperplasia of adipocytes, and these events are regulated by adipocyte differentiation [5]. As such, there is a pressing need to identify the mechanisms and regulators of adipocyte differentiation to discover novel therapies effective at preventing obesity.

Zinc is a major trace element essential for the regulation of various biological processes. As early as 1961, nutritional zinc deficiency was reported to be associated with increased risk of developing multiple diseases including growth retardation, hypogonadism, and anemia; these suggest that zinc may exert its beneficial effects by modulating physiological function [6]. After this seminal finding, zinc was demonstrated to regulate adipocyte metabolism, and serum zinc levels were highly associated with metabolism in obesity. Decreased serum-zinc concentrations were found in subjects with higher body mass index (BMI) values compared to controls [7]. Since this initial report, much interest has been focused on investigating the causal relationship between zinc levels and obesity, but the results remain controversial. There is a growing body of evidence showing the negative association between blood zinc levels and obese status [8,9,10,11,12]. In other words, the higher serum zinc levels, the lower the BMI of subjects in previous studies [9,10,11]. One hypothesis is that adipocytes in obese people may absorb more zinc due to inflammation-induced expression of genes encoding zinc transporters [13,14], leading to altered homeostasis of zinc levels in serum or plasma. However, this hypothesis was not supported by other studies that showed that the level of zinc in the blood may not be correlated with weight, BMI, or waist circumference [15,16,17]. Moreover, intracellular Zn^2+^ concentration may be more important than serum Zn^2+^ in regulating adipocyte development. For example, it was demonstrated that the zinc transporter ZIP14 participates in the uptake of Zn^2+^ during the early stages of adipocyte differentiation [18] and that ZIP14 deficiency causes hypertrophic adipose tissues in mice [19,20]. However, the relationship between intracellular Zn^2+^ levels and adipocyte differentiation are still not clear.

Nitric oxide (NO) is a ubiquitous signaling molecule that is known to directly regulate adipocyte function. The expression of endothelial nitric oxide synthase (eNOS) and inducible nitric oxide synthase (iNOS) genes in adipocytes is significantly increased in obesity [20], which is a chronic inflammatory state. Consequently, clinical observations showed that increases in circulating NO levels strongly correlated with body fat in obesity, an insulin-resistant state [21]. In addition, previous studies showed that biochemical markers of differentiation in primary preadipocytes in response to stimulation with NO promoted lipoprotein lipase- and glycerol-3-phosphate dehydrogenase-specific activities and augmented triglycerides (TG) accumulation [22]. Our previous study found that chronic NO deficiency causes a significant decrease in adipose tissue mass in rats [23]. These findings suggest that decreased NO production could decrease lipid storage in adipose tissues or inhibit adipocyte differentiation. On the other hand, increased NO production may increase lipid storage in adipose tissue or stimulate adipocyte differentiation.

Nitric oxide may regulate intracellular zinc homeostasis in multiple tissues and cell types. Animal studies revealed that NO generators result in the accumulation of zinc in hippocampal neuronal perikarya [24]. Similar results have been found showing that NO strongly increases the amount of labile Zn^2+^ in endothelial cells [25,26], splenocytes [26], and neuroendocrine pheochromocytoma [27]. Collectively, these observations suggest the possibility that in addition to Zn^2+^, NO may act as another stimulus that induces adipocyte differentiation. Yet, in vitro evidence to support this hypothesis is still lacking.

Therefore, we hypothesized that NO stimulates increases in intracellular Zn^2+^, ultimately regulating adipocyte differentiation. The present study tested this hypothesis by studying the effects of Zn^2+^ on NO-mediated adipocyte differentiation and its underlying regulatory mechanisms in 3T3-L1 fibroblasts.

## 2. Results

### 2.1. Effect of ZnCl_2_ on Cell Viability and Adipocyte Differentiation

**Figure 1:**
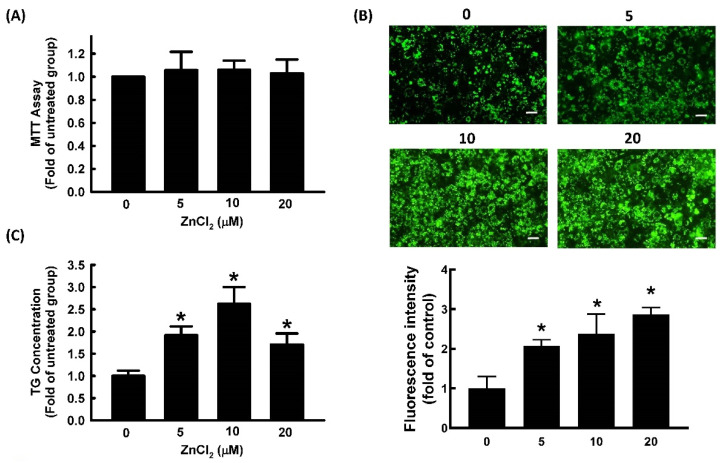
Zinc promotes adipocyte differentiation in 3T3-L1 adipocytes. Adipocytes were cultured in the presence or absence of ZnCl_2_ (0, 5, 10, and 20 μM) to determine its cytotoxicity (**A**), and adipocyte differentiation was induced in the continued presence of the same concentrations of ZnCl_2_ in later experiments. After differentiation, intracellular lipid content was determined by measuring fluorescent staining (Bar: 100 µm) (**B**) and triglycerides (TG) (**C**). Results are shown as the mean ± SD for three independent experiments. * *p* < 0.05 compared to the untreated group.

The 3-[4,5-dimethylthiazol-2-yl]-2,5 diphenyl tetrazolium bromide (MTT) assays are commonly used to evaluate cytotoxicity in various cell models. Different dosages of ZnCl_2_ (0, 5, 10, and 20 μM) were added into 3T3-L1 cell culture media for 72 h, and cellular viability was determined with MTT assays. ZnCl_2_ at concentrations of ≤20 μM showed no significant cytotoxicity in 3T3-L1 cells (Figure 1A). Therefore, 0–20 μM of ZnCl_2_ was used in subsequent experiments. To evaluate the effect of ZnCl_2_ on 3T3-L1 fibroblast differentiation, the TG content of adipocytes was measured. Treatment with 5, 10, or 20 μM of Zn^2+^ increased TG content by 185%, 220%, and 168%, respectively, relative to the vehicle control (Figure 1B). The strongest stimulatory effect of Zn^2+^ on adipocyte differentiation was observed with 10 μM of ZnCl_2_. Lipid accumulation was further measured by BODIPY 493/503 staining; its results showed that ZnCl_2_ increased the accumulation of lipid droplets in a dose-dependent manner (Figure 1C).

**Figure 2:**
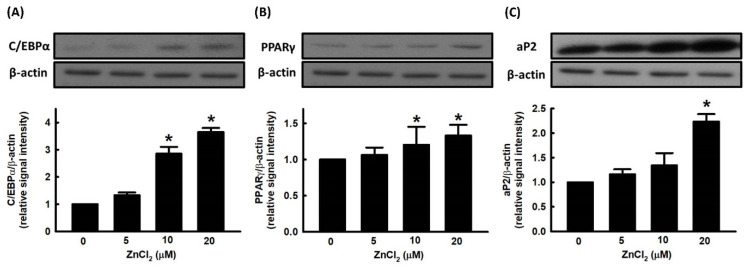
Zinc alters the expression of adipogenic proteins during adipocyte differentiation. 3T3-L1 adipocytes were incubated in the medium in the presence or absence of ZnCl_2_. Adipocyte differentiation was induced, and the expression of adipogenic proteins C/EBPα (**A**), PPARγ (**B**), and aP2 (**C**) was measured by immunoblotting with β-actin as the loading control. Those immunoblots are displayed as examples. Results are shown as mean ± SD for three independent experiments. * *p* < 0.05 compared with the vehicle control.

There are two families of transcription factors involved in the regulation of adipocyte differentiation, including CCAAT enhancer binding proteins (C/EBPs) and peroxisome-proliferator-activated receptor (PPARs). Preadipocytes exposed to inducers of differentiation manifest an early and transient increase in the expression of C/EBPβ and C/EBPδ, which in turn appear to contribute to cell proliferation and a subsequent increase in the expression of C/EBPα and peroxisome-proliferator-activated receptor γ (PPARγ) [28]. We further evaluated the effect of ZnCl_2_ on the expression of adipogenic transcription factors, such as C/EBPα and PPARγ. ZnCl_2_ stimulated the expression of C/EBPα and PPARγ in a dose-dependent manner (Figure 2A,B). We further measured the effect of ZnCl_2_ on the expression of the adipocyte-specific protein, adipocyte protein 2 (aP2), and found that the protein levels of aP2 were significantly increased compared to untreated cells (Figure 2C).

### 2.2. Effect of NO on Cell Viability and Adipocyte Differentiation

**Figure 3:**
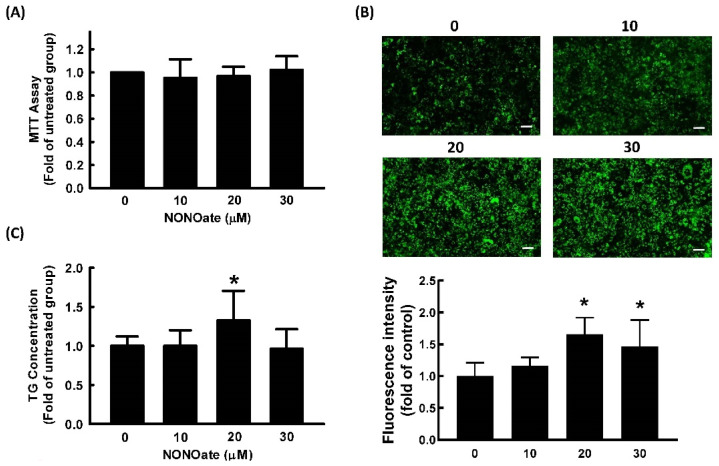
NO promotes adipocyte differentiation in 3T3-L1 adipocytes. Adipocytes were cultured in the presence or absence of NONOate (0, 10, 20, and 30 μM) to determine its cytotoxicity (**A**). Adipocyte differentiation was induced in the continued presence of the same concentrations of NONOate in later experiments. After differentiation, intracellular lipid content was determined by measuring fluorescent staining (Bar: 100 µm) (**B**) and triglycerides (TG) (**C**). Results are shown as mean ± SD for three independent experiments. * *p* < 0.05 compared to the untreated group.

Diethylenetriamine NONOate was used as a NO donor in our study because of its prolonged release of NO due to its long half-life (20 h at pH 7.4, 37 °C) [29]. We first examined the cell viability of 3T3-L1 cells treated with the NO donor, NONOate. The results of the MTT assay showed that NONOate at concentrations ≤30 μM showed no significant cytotoxicity in 3T3-L1 cells (Figure 3A). Therefore, 0–30 μM of NONOate was employed in subsequent experiments.

To investigate the effect of NONOate on adipocyte differentiation, 3T3-L1 fibroblasts were induced to differentiate in the medium in the presence or absence of NONOate, and the lipid content was quantified. As shown in Figure 3B,C, treatment with 20 μM NONOate resulted in a significant increase in triglyceride accumulation compared with the vehicle control.

**Figure 4:**
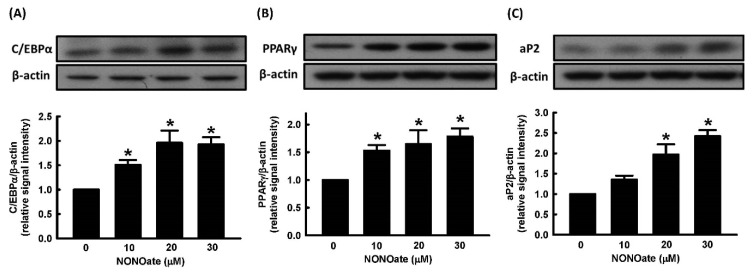
NO alters the expression of adipogenic proteins during adipocyte differentiation. 3T3-L1 adipocytes were incubated in the medium in the presence or absence of NONOate (0, 10, 20, and 30 μM), and adipocyte differentiation was induced in the continued presence or absence of NONOate. The expression of adipogenic proteins C/EBPα (**A**), PPARγ (**B**), and aP2 (**C**), was measured by immunoblotting with β-actin as the loading control. Those immunoblots are displayed as examples. Results are shown as mean ± SD for three independent experiments. * *p* < 0.05 compared with the vehicle control.

We further evaluated the effect of NONOate on the expression of the adipogenic transcription factors, C/EBPα and PPARγ. NONOate stimulated the expression of C/EBPα and PPARγ in a dose-dependent manner (Figure 4A,B). We further measured the effect of NONOate on the expression of aP2 and found that aP2 protein was significantly increased compared to untreated cells (Figure 4C).

### 2.3. Effects of ZnCl_2_ and NO on Intracellular Zn^2+^ Mobilization

**Figure 5:**
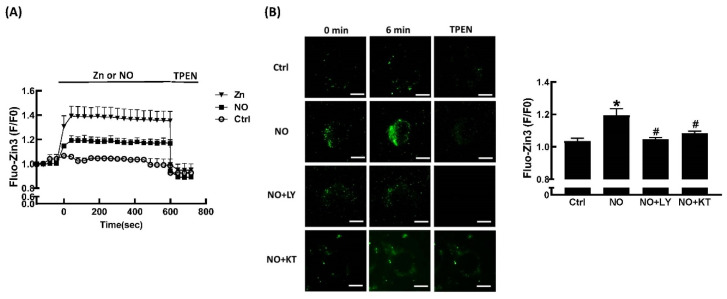
NO stimulates Zn^2+^ mobilization through the GC/cGMP/PKG pathway. Representative traces in the presence or absence of ZnCl_2_ or NONOate (20 μM), which were immediately inhibited by addition of TPEN (10 μM) (**A**). Fluorescence intensity traces were normalized to the signal obtained at time zero and were presented as F/F0. Representative fluorescence images at baseline and 6 min after exposure to 20 μM NONOate with or without GC inhibitor LY83583 (LY; 10 μM) or PKG inhibitor KT5823 (KT; 10 μM) in 3T3-L1 fibroblasts (**B**). NONOate clearly enhanced fluorescence intensity and was reversible with the addition of TPEN (10 μM). Both LY83583 and KT5823 blocked the action of NONOate. Summary data of FluoZin™-3 fluorescence intensity after 6 min of exposure to NONOate with or without LY83583 and KT5823 was expressed as a percentage of baseline. Results are shown as the mean ± SD for three independent experiments. * *p* < 0.05 compared to the untreated group. ^#^ *p* < 0.05 compared to the group treated with NONOate alone.

We used the fluorescent zinc ion indicator FluoZin™-3 AM to study changes in intracellular Zn^2+^ levels in response to extracellular ZnCl_2_ and NONOate. As shown in Figure 5A, no change in Fluo-Zin3 fluorescence over time was seen in untreated cells. In contrast, incubation with ZnCl_2_ for 1 min significantly increased FluoZin-3 fluorescence. Addition of the membrane-permeable Zn^2+^-selective chelator, TPEN, immediately and significantly decreased FluoZin-3 fluorescence to baseline. Incubation with NONOate for 1 min significantly increased FluoZin-3 fluorescence, and addition of TPEN immediately and significantly decreased FluoZin-3 fluorescence to baseline. NO is reported to stimulate guanylate cyclase, which catalyzes the production of cyclic guanosine monophosphate (cGMP). Subsequently, this synthetic cGMP activates downstream protein kinase G (PKG) [30]. LY83583, an inhibitor of guanylate cyclase (GC) and of cGMP production, was used to investigate the mechanisms mediating NONOate-induced Zn^2+^ mobilization. LY83583 prevented the NONOate-dependent increase in FluoZin-3 fluorescence (Figure 5B), suggesting that GC/cGMP is responsible for the mobilization of Zn^2+^ by NO. In order to further clarify the role of PKG in NONOate-induced Zn^2+^ mobilization, KT5823, a specific membrane-permeable PKG inhibitor, was utilized. As shown in Figure 5B, pretreatment with KT5823 significantly prevented the NONOate-induced increase in FluoZin-3 fluorescence, indicating that activation of PKG may contribute to the Zn^2+^-releasing effect of NO.

### 2.4. Role of Zn^2+^ in NO-Stimulated Adipocyte Differentiation

**Figure 6:**
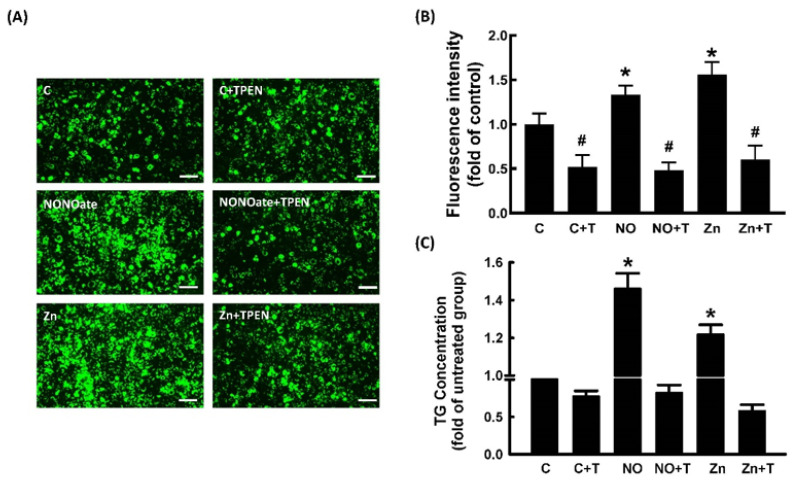
NO-stimulated adipocyte differentiation in 3T3-L1 adipocytes was Zn^2+^-dependent. Adipocytes were incubated in medium in the presence or absence of TPEN (10 μM) with or without NONOate (20 μM) or ZnCl_2_ (20 μM), and adipocyte differentiation was induced. After differentiation, intracellular lipid content was determined by measuring fluorescent staining (**A**,**B**) and triglycerides (TG) (**C**). Results shown as mean ± SD for three independent experiments. * *p* < 0.05 compared to the untreated group. ^#^ *p* < 0.05 compared to matched sets of the TPEN-untreated group.

**Figure 7:**
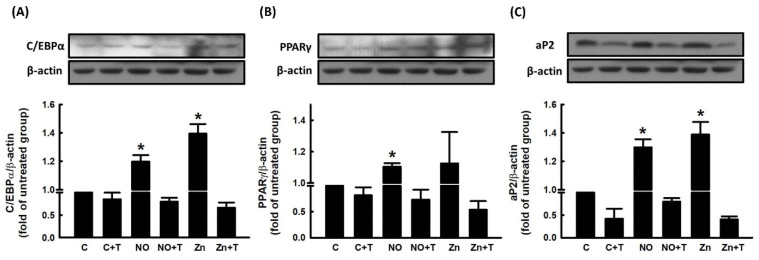
NO alters the expressions of adipogenic proteins during adipocyte differentiation via a Zn^2+^-dependent pathway. 3T3-L1 adipocytes were incubated in the presence or absence of TPEN (10 μM) with or without NONOate (20 μM) or ZnCl_2_ (20 μM), and adipocyte differentiation was induced. The expression of the adipogenic proteins C/EBPα (**A**), PPARγ (**B**), and aP2 (**C**) was measured by immunoblotting with β-actin as the loading control. Those immunoblots are displayed as examples. Results are shown as mean ± SD for three independent experiments. * *p* < 0.05 compared to the untreated group.

We further explored the role of Zn^2+^ in NO-stimulated adipocyte differentiation. To examine whether Zn^2+^ was involved in NO-stimulated adipocyte differentiation, pretreatment with TPEN was used to block Zn^2+^ release in 3T3-L1 fibroblasts. As shown in Figure 6, NONOate- and Zn^2+^-dependent increases in triglyceride accumulation were significantly suppressed by TPEN treatment. The effect of TPEN on the expression of NONOate-stimulated and Zn^2+^-stimulated adipogenic factors was measured by immunoblotting. NONOate and Zn^2+^ treatments both increased the expression of aP2, PPARγ, and C/EBPα compared with controls, and both NONOate-upregulated and Zn^2+^-upregulated C/EBPα, PPARγ, and aP2 were significantly suppressed by TPEN treatment (Figure 7A–C). These findings support the possibility that NONOate-stimulated adipocyte differentiation is Zn^2+^-dependent. In addition, GC inhibitor LY83583 and PKG inhibitor KT 5823 prevented the NONOate-dependent increase in intracellular Zn^2+^ mobilization (Figure 5); they also suppressed NONOate-dependent increases in triglyceride accumulation in 3T3-L1 adipocytes (Supplementary Figure S1).

### 2.5. Correlation between Adipocyte Size, Zn^2+^ Level, and NOS Expression in Human Adipose Tissue

**Figure 8:**
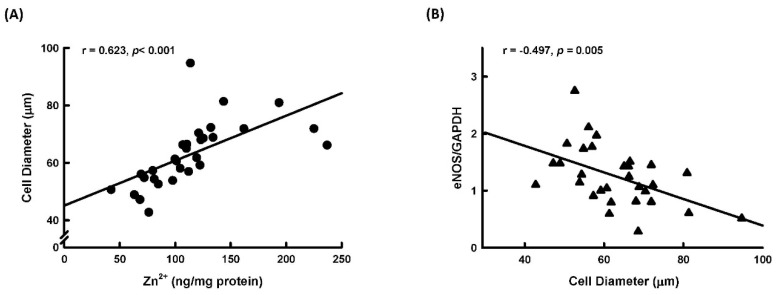
Correlation analysis between human adipocyte size and tissue Zn^2+^ levels or eNOS expression. Human adipocyte diameter was positively correlated with levels of tissue Zn^2+^ (**A**) and was negatively correlated with eNOS expression (**B**).

We further explored the relationship between adipocyte size, tissue Zn^2+^ levels, and expression of iNOS and eNOS in normal human subcutaneous tissue. As shown in Figure 8A, adipocyte size was positively correlated with tissue Zn^2+^ levels. This correlation is in agreement with the findings that Zn^2+^ could stimulate adipocyte differentiation. Additionally, adipocyte size was negatively correlated with expression of eNOS (Figure 8B), but there was no correlation between adipocyte size and iNOS expression (r = 0.05527, *p* = 0.77203).

## 3. Discussion

In this study, we demonstrated that the addition of extracellular Zn^2+^ could increase intracellular Zn^2+^ concentration and stimulate adipocyte differentiation. Furthermore, we found that adipocyte size positively correlated with tissue Zn^2+^ levels in human subcutaneous fat tissue. Zinc is an essential trace element for all living organisms. Zn^2+^ is necessary for the structure and function of Zn^2+^-binding protein, and it acts as an intracellular signaling molecule, regulating various cellular functions. Two families of Zn^2+^ transporter proteins regulate cellular zinc homeostasis, including the Zn^2+^ transporter (ZnT) family, which controls Zn^2+^ efflux out of the cytosol, and the Zrt/Irt-related protein (ZIP) family, which controls Zn^2+^ influx into the cytosol. Dysregulated zinc signaling leads to pathophysiological disturbances [31]. Additionally, intracellular Zn^2+^ deficiency significantly reduces the DNA-binding activity of PPARγ and impairs PPARγ signaling [32]. It was speculated that Zn^2+^ may regulate PPARγ function and affect adipocyte differentiation. Furthermore, several zinc finger proteins were reported to regulate adipocyte differentiation [33]. Nguyen’s study demonstrated that overcharging of Zn^2+^ in the structure of the zinc finger protein Is needed for DNA binding stability [34]. Therefore, intracellular Zn^2+^ deficiency may affect the structure of the zinc finger protein and lead to DNA binding instability. Collectively, intracellular Zn^2+^ levels are important for adipocyte differentiation; our present study demonstrated that elevation of intracellular Zn^2+^ levels could stimulate adipocyte differentiation in a dose-dependent manner.

To our knowledge, our study is the first to demonstrate that NO could regulate intracellular Zn^2+^ mobilization in adipocytes. These results were comparable to a study by Hung and coworkers in cultured rat embryonic cortical neurons [35]. In their study, they demonstrated that an inhibitor of neuronal NO synthase (vinyl-_L_-NIO) significantly suppresses the dopamine-induced elevation of intracellular Zn^2+^ concentration, and that NO generators like NONOate increase intracellular Zn^2+^ concentrations in cultured neurons [35]. Additionally, NO-induced mobilization of intracellular Zn^2+^ was demonstrated in isolated cardiomyocytes [30]. NO-mediated zinc release was also observed in mouse lung endothelial cells [36]. Taken together with our findings, NO may modulate intracellular Zn^2+^ mobilization to regulate diverse biological functions. Moreover, we further demonstrated that NO stimulated intracellular Zn^2+^ mobilization in adipocytes through the GC/cGMP/PKG pathway (Figure 5). These results were also comparable to Jang’s study in isolated rat cardiomyocytes [35].

Our observations in human subcutaneous fat tissue found that eNOS expression was negatively correlated with adipocyte size. This finding was comparable to Razny’s and Sansbury’s findings that genes associated with adipogenesis were upregulated in eNOS-deficient mice [37] and that overexpression of eNOS could prevent high-fat diet-induced obesity in eNOS transgenic mice [38]. Previous studies demonstrated that expression of eNOS and iNOS in adipocytes is significantly increased in obesity [20] and that iNOS expression is significantly increased during adipocyte differentiation [20,22]. Our previous study found that chronic NO deficiency causes a significant decrease in adipose tissue mass in rats [23]. In human mesenchymal stem cells, endothelial NO synthase knockdown blocks adipogenesis [39]. On the other hand, Jang’s study demonstrated that macrophage-derived NO could inhibit adipocyte differentiation [40]. Furthermore, overexpression of endothelial NO synthase prevented diet-induced obesity [38]. Results of a stem cell differentiation study showed that blocking endogenous NO synthase significantly stimulated adipogenic differentiation, whereas treatment with a NO donor significantly reduced adipogenic differentiation [41]. Collectively, the role of NO in regulating adipocyte differentiation is controversial. In the present study, we demonstrated that NO could stimulate adipocyte differentiation and that the underlying mechanism was Zn^2+^-dependent.

Zn^2+^ plays a critical role in the process of adipocyte differentiation. Zn^2+^ homeostasis is perturbed in the pathogenesis of diabetes, and inadequate Zn^2+^ distribution may affect the onset of diabetes and metabolic diseases by regulating various critical biological events [42]. A recent study demonstrated that a Zn^2+^ transporter deficient mouse (Zip13) had enhanced beige adipocyte biogenesis and energy expenditure and displayed ameliorated diet-induced obesity and insulin resistance [43]. Adipose tissue from Zip14 knockout mice had increased levels of preadipocyte markers and lower expression of differentiation markers compared with wild-type controls [19]. However, the association between the expression of zinc transporters and NO synthase, NO production, and intracellular Zn^2+^ concentration in obesity is still not clear.

**Figure 9:**
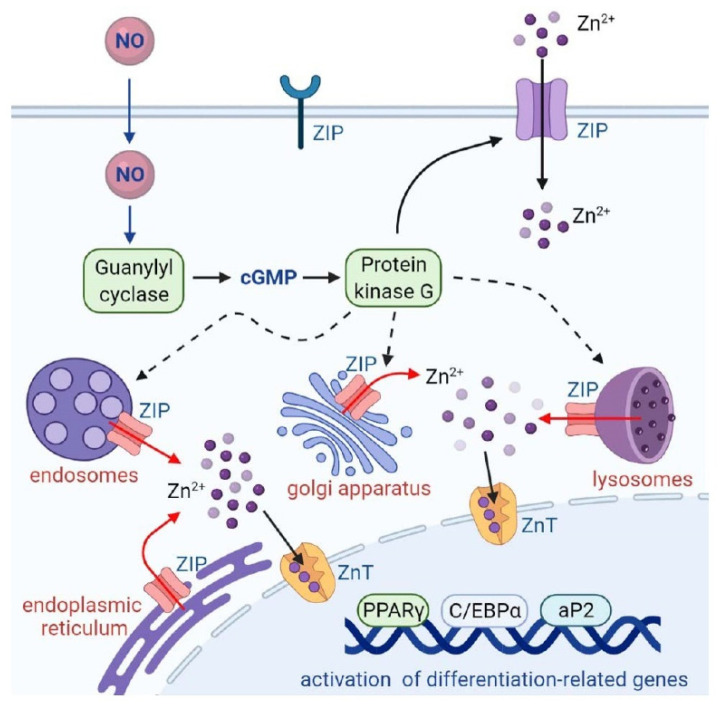
Schematic summary. NO mobilizes intracellular zinc and stimulates adipocyte differentiation. NO stimulates GC/cGMP/PKG signaling to mobilize intracellular zinc and promotes the expression of differentiation signaling proteins (C/EBPα, PPARγ, and aP2), leading to adipocyte differentiation.

In conclusion, our data demonstrated that NO treatment stimulated intracellular Zn^2+^ mobilization through the GC/cGMP/PKG pathway, caused upregulation of adipocyte differentiation regulators such as PPARγ, C/EBPα, and aP2, and resulted in triglyceride accumulation in 3T3-L1 adipocytes (Figure 9). The impact of zinc on obesity and its associated metabolic disorders has been shown in a study by Nasab and coworkers [44]. Their data showed that urinary Zn^2+^ concentrations are greater in obese adults and that zinc levels are significantly associated with fasting blood sugar and lipid metabolites including cholesterol, triglyceride, LDL, and HDL. Our findings suggest that abnormal NO production may interfere with the homeostasis of intracellular Zn^2+^, stimulating adipocyte differentiation and resulting in the development of obesity.

## 4. Material and Methods

### 4.1. Materials

The 3T3-L1 fibroblast cell line was obtained from American Type Culture Collection (Rockville, MD, USA). Dulbecco’s modified Eagle medium (DMEM), penicillin, and streptomycin were purchased from Gibco BRL (Gaithersburg, MD, USA). Fetal bovine serum was obtained from Biowest (Nuaillé, France). Isobutylmethylxanthine (IBMX), dexamethasone, and all other chemicals were obtained from Sigma-Aldrich (St. Louis, MO, USA). Antibodies targeting C/EBPα, PPARγ, and α-tubulin were purchased from Santa Cruz Biotechnology (Santa Cruz, CA, USA). Antibodies against aP2 were purchased from Chemicon International (Temecula, CA, USA). The triglyceride assay kit was purchased from DiaSys Diagnostic Systems GmbH (Holzheim, Germany).

### 4.2. Experimental Design

We first checked cell viability in response to various dosages of ZnCl_2_ or the NO donor, NONOate, to determine the optimal dosage ranges for follow-up experiments. To explore the effects of Zn^2+^ and NO on adipocyte differentiation, post-confluent 3T3-L1 fibroblasts were pretreated with ZnCl_2_ or NONOate, followed by incubation with differentiation inducers. The accumulation of intracellular lipids, concentration of triglyceride, and expression of adipocyte-differentiation-related transcription factors and adipocyte-specific genes was measured after differentiation. To explore the effects of Zn^2+^ and NO on intracellular Zn^2+^ mobilization, 3T3-L1 fibroblasts were preloaded with FluoZin™-3 AM for 30 min, and ZnCl_2_- or NONOate-induced intracellular zinc signals were measured. To clarify the involvement of GC/cGMP/PKG signal cascades in NO-induced Zn^2+^ mobilization, 3T3-L1 fibroblasts were pretreated with the GC inhibitor, LY83583, or PKG inhibitor, KT 5823, for 30 min, and NONOate-induced intracellular zinc signals were measured. To further elucidate the role of Zn^2+^ in NO-stimulated adipocyte differentiation and intracellular Zn^2+^ mobilization, cells were preincubated with a membrane-permeable Zn^2+^ chelator (TPEN) for 1 h, followed by incubation with NONOate. The efficiency of adipocyte differentiation and intracellular Zn^2+^ mobilization was measured. The relationship between adipocyte size, Zn^2+^ levels, and NOS expression was further explored by using normal subcutaneous fat tissue from patients in breast cancer surgeries. Adipocyte size was examined by using immunohistochemistry. Tissue Zn^2+^ levels were measured by NexION 350 ICP-MS (PerkinElmer, Inc., Shelton, CT, USA). NOS expression in adipose tissue was evaluated by using real-time RT-PCR.

### 4.3. 3T3-L1 Fibroblast Cell Culture and Differentiation Conditions

3T3-L1 fibroblasts (American Type Culture Collection, Rockville, MD, USA) were seeded into six-well cell culture plates (Falcon, Becton Dickinson, NJ, USA) and were grown and maintained in DMEM containing 100 units/mL penicillin, 100 μg/mL streptomycin (both from Gibco BRL, Gaithersburg, MD, USA), and 10% fetal bovine serum (Biowest, Nuaillé, France) (complete medium) in 10% CO_2_. About 3 × 10^5^ cells/well were seeded into six-well cell culture plates. Cell number at confluence was about 1.2 × 10^6^ cells/well. Cells were grown to 2 days post-confluency and were differentiated by incubating them for 3 days in complete medium containing isobutylmethylxanthine (IBMX; 0.5 mM), dexamethasone (0.5 μM), and insulin (1.7 μM) (all from Sigma, St. Louis, MO, USA). Cells were then maintained in complete medium containing 10% insulin for another three days. The medium was changed every three days until the cells were fully differentiated. Typically, by day 10, more than 95% of the fibroblasts had differentiated into mature adipocytes as determined by staining for lipid accumulation using BODIPY 493/503.

### 4.4. BODIPY 493/503 Staining

To examine lipid accumulation, cells cultured in 12-well plates were fixed with formalin and stained with BODIPY 493/503. For photomicrographs, cells were counterstained with DAPI. BODIPY 493/503 is an alternative lipid dye with low background staining and a narrow emission spectrum.

### 4.5. Measurement of Triglyceride

Intracellular triglyceride content was measured by a colorimetric method using triglyceride assay kits (DiaSys Diagnostic Systems GmbH, Holzheim, Germany).

### 4.6. Immunoblot Analysis

Whole cell lysates were collected by sonication in lysis buffer (1% Triton X-100, 50 mM KCl, 25 mM Hepes, pH 7.8, 10 µg/mL leupeptin, 20 µg/mL aprotinin, 125 µM dithiothreitol, 1 mM phenylmethylsulfonyl fluoride) containing protease and phosphatase inhibitor cocktails. Samples (100 µg of total protein) in 50 µL of Laemmli sample buffer were boiled for 10 min and resolved with 15% mini-SDS-PAGE. The contents of the gel were then transferred onto a polyvinylidene difluoride membrane. The membrane was pre-blotted in 5% skim milk in phosphate-buffered saline (PBS) for 60 min at room temperature and then immunoblotted with the indicated primary antibodies overnight at 4 °C, followed by 60 min labeling with a secondary antibody conjugated with horseradish peroxidase at room temperature. After the chemiluminescence reaction (Amersham Biosciences, Buckinghamshire, UK), bands were detected by exposing blots to X-ray films in a dark environment for an appropriate period of time.

### 4.7. MTT Assay

Cells were incubated in 12-well plates for 72 h, with or without NO/Zn^2+^. The medium was replaced with a medium containing 1 mg/mL MTT solution (USB, Amersham Life Sciences, Cleveland, OH, USA) and cells were incubated for 2 h. The supernatant was removed, and 100 μL of dimethyl sulfoxide (DMSO, Sigma-Aldrich Chemical Company, St. Louis MO, USA) was added to each well to fully dissolve the formazan crystals that were produced inside the live cells. The absorbance was measured with a microplate reader at 570/630 nm.

### 4.8. Live-cell Imaging of Intracellular Zinc

Microscopy was conducted with a Zeiss LSM880 confocal microscope. 3T3-L1 fibroblasts were preloaded with 5 μM FluoZin™-3 AM, diluted in cell imaging medium (phenol red free DMEM media supplemented with 10% FBS), for 30 min. Cells were washed three times with PBS and 1 mL of cell imaging medium was added. The excitation wavelength used was 485 nm, and the emission wavelength was 510–540 nm. Intensities of intracellular zinc signals in live cells were measured from 10 pre-chosen cytosolic regions (1 μm^2^) per cell, and the mean value for each cell was obtained.

### 4.9. Collection of Human Subcutaneous Adipose Tissues

Thirty female breast cancer patients were recruited from one outpatient department of the Taipei Veterans General Hospital in Taiwan. Normal subcutaneous adipose tissue weighing about 5 g was obtained from patients in breast cancer surgeries. Adipocyte size was examined by using immunohistochemistry. Tissue Zn^2+^ levels were measured by NexION 350 ICP-MS (PerkinElmer, Inc., Shelton, CT, USA). NOS expression in adipose tissue was evaluated by using real-time RT-PCR. The protocol was reviewed and approved by the Institutional Review Board of the National Yang Ming Chiao Tung University (YM104146EF). Patients were only entered into the study after informed written consent had been obtained.

### 4.10. Real-time Polymerase Chain Reaction (RT-PCR)

Total RNA was extracted by using a Tri Reagent kit (Sigma-Aldrich, St. Louis, MO, USA), and the extraction was carried out as previously described [45]. TaqMan Gene Expression Assays (FAM dye-labeled MGB probe; Applied Biosystems, Foster City, CA, USA) containing specific primers, TaqMan MGB probe (FAM dye-labeled), TaqMan Fast Universal PCR Master Mix, and 100 ng of cDNA were used to detect and quantify mRNA expression. The probe was obtained from Thermo Fisher (Thermo Fisher, Inc., Waltham, MA, USA); the primers used were iNOS (Hs01075529_m1) and eNOS (Hs01574665_m1). GAPDH mRNA was amplified as the internal control, and GAPDH Ct values were subtracted from those of target genes. Reactions were performed as follows: 95 °C for 10 min followed by 40 cycles at 95 °C for 15 s/60 °C for 1 min.

### 4.11. Statistical Analysis

Statistical analyses were performed using SPSS software (IBM Corp. Armonk, NY, USA). Experiments were repeated at least three times. All results are expressed as mean ± SD. Statistical significance was assessed by one-way analysis of variance or Student’s t test. The correlations between variables were evaluated using Pearson correlation. A value of *p* < 0.05 was considered statistically significant.

## Data Availability

The data used to support the findings of this study are available from the corresponding author upon request.

## Supplementary Materials

The following supporting information can be downloaded at: https://www.mdpi.com/article/10.3390/ijms23105488/s1.
