# Supplementary material for: Nitric Oxide Mobilizes Intracellular Zn2+ via the GC/cGMP/PKG Signaling Pathway and Stimulates Adipocyte Differentiation
Source: Int J Mol Sci. 2022 May 14;23(10):5488. doi: 10.3390/ijms23105488 (PMC9143299; doi:10.3390/ijms23105488)
Supplement: Supplementary file 1 [file ijms-23-05488-s001.zip › ijms-1679010-supplementary.pdf]

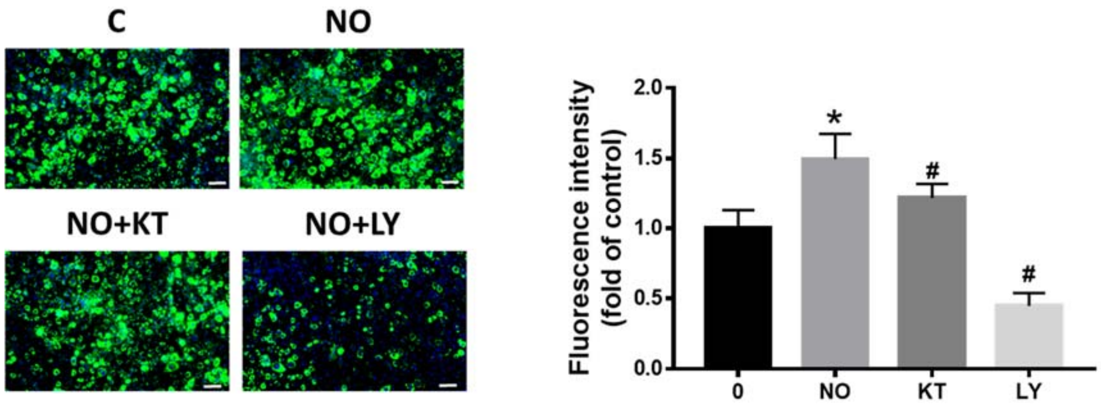

Figure S1. Inhibition of GC/cGMP/PKG pathway significantly suppressed NONOate-dependent increases in triglyceride accumulation in 3T3-L1 adipocytes. (Bar: 100 μm)
